# Oocyte activation is a cytoplasm-confined event so far: what about the nucleus?

**DOI:** 10.1530/REP-23-0360

**Published:** 2024-02-02

**Authors:** Luisa Gioia, Luca Palazzese, Marta Czernik, Domenico Iuso, Helena Fulka, Josef Fulka Jr, Pasqualino Loi

**Affiliations:** 1 Department of Bioscience and Technology for Food, Agriculture and Environment, University of Teramo, Teramo, Italy; 2 Department of Veterinary Medicine, University of Teramo, Teramo, Italy; 3 Institute of Experimental Medicine of the Czech Academy of Sciences, Prague, Czech Republic, Institute of Animal Science, Prague, Czech Republic

## Abstract

The fertilizing spermatozoa induce a Ca^2+^ oscillatory pattern, the universal hallmark of oocyte activation, in all sexually reproducing animals. Assisted reproductive technologies (ARTs) like intracytoplasmic sperm injection (ICSI) bypass the physiological pathway; however, while a normal Ca^2+^ release pattern occurs in some species, particularly humans, artificial activation is compulsory for ICSI-fertilized oocytes to develop in most farm animals. Unlike the normal oscillatory pattern, most artificial activation protocols induce a single Ca^2+^ spike, undermining proper ICSI-derived embryo development in these species. Curiously, diploid parthenogenetic embryos activated by the same treatments develop normally at high frequencies and implant upon transfer in the uterus. We hypothesized that, at least in ruminant embryos, the oscillatory calcium waves late in the first cell cycle target preferentially the paternal pronucleus and are fundamentally important for paternal nuclear remodeling. We believe that Ca^2+^ signaling is central to full totipotency deployment of the paternal genome. Research in this area could highlight the asymmetry between the parental genome reprogramming timing/mechanisms in early development and impact ARTs like ICSI and cloning.

## Background and hypothesis

The fertilizing spermatozoon sparks a series of Ca^2+^ oscillations that activate the transition of the quiescent egg into an embryo (Swann 2022). As the cell most subjected to micromanipulations, commonly defined as assisted reproductive technologies (ARTs), the normality of the oocyte *in vitro* fertilization event is often subverted. ARTs are typically used in laboratory and farm animals and routinely in human infertility treatments (Herbert *et al.* 2023). ARTs come with various levels of invasiveness, ranging from the simple intracytoplasmic sperm injection (ICSI) to cloning, where a more or less differentiated nucleus is injected into an oocyte deprived of its own chromosomes (Czernik *et al.* 2019). As these procedures bypass physiological activation, artificial activation is compulsory (Briski & Salamone 2022). Physical, chemical, and mechanical approaches are being used to increase the intracellular levels of Ca^2+^ in the oocyte (Stein *et al.* 2020). However, data on Ca^2+^ mobilization and the number of spikes and their persistence following ARTs are scarce. Our recent data on sheep embryo development following ICSI indicated that the standard treatments typically induce a single Ca^2+^ pulse, with the return to baseline thereafter (Gioia L., unpublished observations). A 30-year retrospective analysis of ART results, including data obtained in our laboratory, where ICSI and cloning are routinely performed, advanced few reflections. It has been proved that artificial activation mirrors the physiological one only partially and varies according to the manipulation type carried out on the oocyte. Interspecies differences also play an important role. While human, mouse, and horse oocytes (Briski & Salamone 2022) respond positively with a normal activation following ICSI, other farm animals do not. Species-specific differences in the presence, activation, and disappearance of phospholipase C zeta could account for defective pathway activation (Stein *et al.* 2020). However, even artificial activation cannot save poor ICSI responders, like sheep, pigs, goats, and cattle, where embryo development to the blastocyst stage remains very poor (Salamone *et al.* 2017), overlapping the poor development recorded following the artificial activation in human and mouse oocytes following the injection of round spermatids (Yanagida *et al.* 2008). This observation clashes with the observation that activated oocytes carrying a diploid set of chromosomes (parthenogenetic development) display normal pre- and post-implantation development in all species (Loi *et al.* 1998). Why is one Ca^2+^ pulse sufficient to unfold the development potential of a parthenogenetic embryo but is very limited following ICSI? We hypothesized that multiple Ca^2+^ waves are fundamentally required only when two pronuclei are present in the zygote and that one of them was from the male.

Our point of view suggests a functional asymmetry between male and female nuclear reprogramming. It highlights the need for additional post-gametic efforts on the paternal chromosome set brought in by the spermatozoon, given that the maternal one is fully reprogrammed during oocyte growth (Fulka *et al.* 2023). If we activate excellent quality oocytes, such as *in vivo* ovulated ones, all will reach the blastocyst stage and implant following transfer into the uterus, just like normal conceptuses (Loi *et al.* 1998). These findings unequivocally demonstrate that the maternal genome has undergone extensive reprogramming during oocyte growth, further input is not required, and it can drive normal pre- and post-embryonic development, depending on the limits imposed by genomic imprinting.

**Figure 1:**
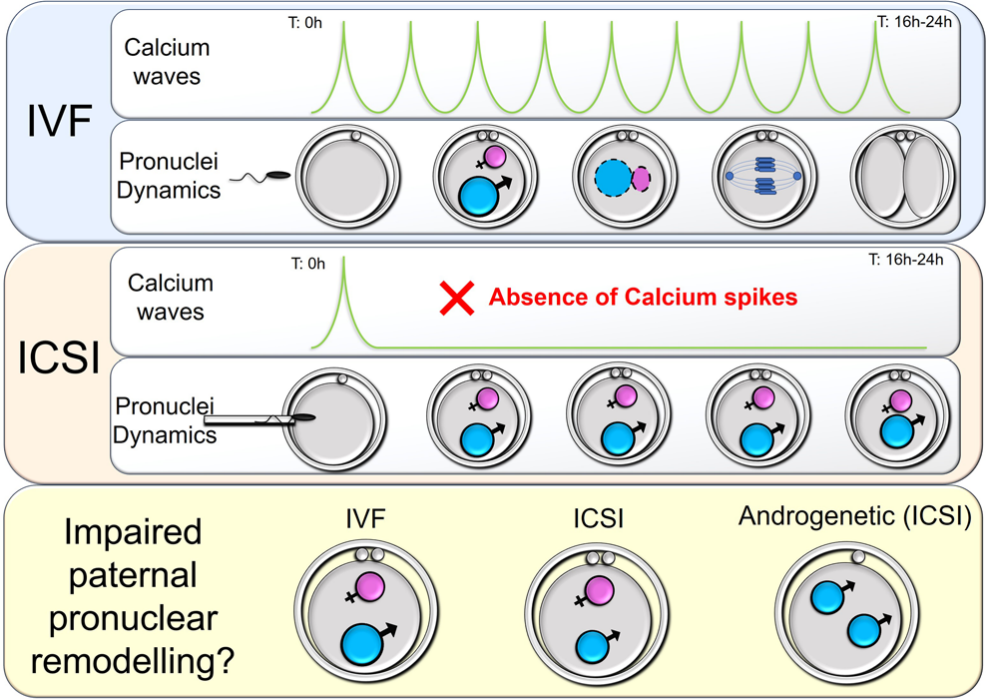
Ca^2+^ and nuclear dynamics in sheep IVF (upper row) and ICSI (middle row) oocytes. The paternal pronucleus size in ICSI embryo is smaller than the IVF one, particularly in androgenetic zygotes (bottom row).

Defective nuclear remodeling appears very early in development and is evident by the much smaller paternal pronuclear in ICSI-derived zygotes than in normal IVF ones (pronuclear area of 354 and 444 μm^2^, respectively; L Palazzese, unpublished data; Fig. 1A). Androgenetic embryos produced by injecting two spermatozoa into enucleated sheep oocytes showed two small-sized pronuclei (Fig. 1B).

Impaired embryo development after ICSI in sheep is not due to insufficient reprogramming; even if this were the case, it is not the primary cause. The lack of calcium spikes during zygotic development jeopardizes the migration and subsequent apposition of the pronuclei. All these events are unequivocally calcium dependent; therefore, it is not surprising that over 85% of activated ICSI zygotes remain in the pronuclear stage (Ressaissi *et al.* 2021). Thus, the lack of syngamy activates the strong G2M block operative in oocytes. Still, the significant differences in pronuclear swelling are strong indicators of reduced remodeling in the paternal pronuclei. Remodeling of the paternal pronucleus occurs in two steps: a rapid one, mediated by nucleoplasm removal of protamine, and a subsequent chromatin reconstitution phase using the maternal histone pool (Inoue *et al.* 2011). Next, ATP-dependent chromatin remodeling engines bound to the chromatin induce and maintain the open structure typical of embryonic nuclei (Crodian *et al.* 2019). While the role of calcium in the cytoplasm is well defined, little is known about its influence on the nuclear compartment. The lack of data on nuclear calcium signaling during mammalian embryo development contrasts the consolidated knowledge of the important role calcium plays in plant nuclei (Mazars *et al.* 2011). Initially met with skepticism, several studies have converged on the evidence that plant cell nuclei possess independent Ca^2+^ stores and hence their own Ca^2+^ homeostasis. Whether alone or in association with other messengers, Ca^2+^ regulates gene expression (Mazars *et al.* 2011).

The situation is not yet defined in embryos, although it is coming of age. Studies in human oocytes and early embryos have identified an important calcium storage protein, calreticulin, and the canonical calcium release receptor, InsP3 (Balakier *et al.* 2002). However, this is only the tip of the iceberg. It has long been established that artificial manipulations, i.e. increasing or decreasing the number of calcium spikes in the first cell cycle in mouse oocytes, profoundly affected gene expression patterns in the resulting embryos, particularly after implantation (Ozil *et al.* 2006). This was the first, and so far, only observation of the profound effect elicited by calcium on pre- and post-implantation embryo development. Nuclear calcium homeostasis/signaling has not been described in embryos; however, a closer look at the core molecular mechanism regulating nuclear reprogramming changes our perception. SWI and SNF (SWItch and Sucrose Non-Fermentable) are ATP-dependent nuclear remodeling complexes discovered in yeast (Neigeborn & Carlson 1984). The genes encoding SWI/SNF form a large complex that includes several polypeptides, suggesting large-scale nucleosome rearrangements that facilitate access to transcription factors to enhance gene expression. BAF (SWI/SNF-A) and ‘polybromo-associated BAF,’ also described as PBAF (SWI/SNF-B), are human analogs of the yeast SWI/SNF (Lai *et al.* 2009). However, SWI/SNF-like BAF recruitment is insufficient for chromatin remodeling; a second calcium/calmodulin (CaM)-dependent event is also required.

Clearly, we have not yet scientific evidence that SWI/SNF specifically addresses the paternal pronucleus. However, there is already ample evidence that SWI/SNF is a crucial remodeling engine, given that maternal mutation of *Brg1*, which encodes a catalytic subunit of SWI/SNF, abolishes Zygotic Genome Activation and induces two-cell arrest (Bultman *et al.* 2006). We simply base our speculation on the fact that sheep parthenogenetic embryos, where no calcium release is observed, progress normally during pre- and post-implantation development.

We think that the two parental genomes joined at fertilization have different degrees of totipotency. The maternal one is fully reprogrammed because normal parthenogenetic embryo and fetal development occur even after a single Ca^2+^ input. The paternal gamete is fully remodeled for easy transport through the female reproductive tract but only partially reprogrammed for totipotency. Full reprogramming is elicited only during the first cell cycle(s) once the pronuclei are organized and calcium oscillations activate the major nuclear remodeling engines, as we have suggested. These confer a full open chromatin structure that endows the paternal genome with full totipotency. The role of calcium oscillation in the cytoplasm following activation has been thoroughly investigated (Swann & Lai 2013). Still, nothing is known about their effects on the nuclear compartment in mammalian development. We suggest that nuclear calcium release is essential for chromatin remodeling, which is driven by calcium-activated, ATP-dependent remodeling engines.

Our hypothesis provides a broader reading frame of calcium signaling in early development that includes the nuclear compartment. Bringing it forward could stimulate further research whose outcomes could bring technological advancements in ARTs, including ICSI.

## Declaration of interest

The authors declare that there is no conflict of interest that could be perceived as prejudicing the impartiality of the study reported.

## Funding

This work was supported by funding from the European Union’s Horizon 2020 Research and Innovation Program under the Marie Skłodowska-Curie action, project “WhyNotDry” (GA-101131087) to PL, MC, and DI; MIUR, PRIN 2022CSHPAS and PNRR P2022FA79R to PL. LP and MC acknowledge support from the National Science Centre, Poland, through grant no. 2016/21/D/NZ3/02610 (Sonata) and 2019/35/B/NZ3/02856 (Opus).

## Author contribution statement

PL, LP, and DI discussed the outline of the manuscript; LG, MC, and LP performed the experiments supporting it; HF and JF provided criticism on the manuscript and edited it; PL wrote the manuscript; and LP prepared the figures.
